# Behavioral Cues of Humanness in Complex Environments: How People Engage With Human and Artificially Intelligent Agents in a Multiplayer Videogame

**DOI:** 10.3389/frobt.2020.531805

**Published:** 2020-11-13

**Authors:** Stephanie Tulk Jesso, William G. Kennedy, Eva Wiese

**Affiliations:** ^1^Social and Cognitive Interactions Lab, Department of Psychology, George Mason University, Fairfax, VA, United States; ^2^Center for Social Complexity, Department of Computational Data Science, College of Science, George Mason University, Fairfax, VA, United States

**Keywords:** Social AI, Human-AI Interactions, Cognitive plausibility, Turing Tests, Social Robotics, Videogames, Virtual Agents

## Abstract

The development of AI that can socially engage with humans is exciting to imagine, but such advanced algorithms might prove harmful if people are no longer able to detect when they are interacting with non-humans in online environments. Because we cannot fully predict how socially intelligent AI will be applied, it is important to conduct research into how sensitive humans are to behaviors of humans compared to those produced by AI. This paper presents results from a behavioral Turing Test, in which participants interacted with a human, or a simple or “social” AI within a complex videogame environment. Participants (66 total) played an open world, interactive videogame with one of these co-players and were instructed that they could interact non-verbally however they desired for 30 min, after which time they would indicate their beliefs about the agent, including three Likert measures of how much participants trusted and liked the co-player, the extent to which they perceived them as a “real person,” and an interview about the overall perception and what cues participants used to determine humanness. *T*-tests, Analysis of Variance and Tukey's HSD was used to analyze quantitative data, and Cohen's Kappa and χ^2^ was used to analyze interview data. Our results suggest that it was difficult for participants to distinguish between humans and the social AI on the basis of behavior. An analysis of in-game behaviors, survey data and qualitative responses suggest that participants associated engagement in social interactions with humanness within the game.

## Introduction

The concept of Artificial Intelligence (AI) is not new. Alan Turing, the father of computer science, predicted that truly “intelligent” machines would appear around the year 2000 (Turing, 1950). According to Google, global leader in AI technology, Explore History of Machine Learning Machine Learning (ML) has become deeply interwoven into our society since the early 2000s. Advances in deep learning have produced near human-level performance in image and speech recognition (LeCun et al., 2015); recent algorithms have even surpassed human world champions in complex competitive games like Go (Silver et al., 2017) and Starcraft II (Vinyals et al., 2019). Still, there is the societal fear that AI will be used in ways that are detrimental to the general public (Piper, 2019). Elon Musk, engineer and entrepreneur, has compared the creation of advanced and unregulated AI to “summoning a demon” (McFarland, 2014). Some benevolent AI creators have used the technology to protect rainforests (Liu et al., 2019), or create diagnostic algorithms that can detect breast cancer better than human experts (McKinney et al., 2020). Other applications can produce undesirable consequences for the general public, such as job loss as a result of automation (Reisinger, 2019), or racial discrimination resulting from biased algorithms used by the U.S. criminal justice system (Angwin et al., 2016).

But beyond traditional applications where AI/ML is used as a tool, the emergence of social AI that attempts to understand and communicate with people in social contexts raises many logistical and ethical questions. It is certainly possible that fears about AI reflect underlying insecurities about human relationships in our present society (Cassell, 2019), especially since technology is developed and applied by human actors who have their own (good and bad) motivations. Advanced robots and AI that are designed to be “social agents” that can interact with humans in meaningful and socially intelligent ways might provide great benefit to humans when applied in human-centric fields like healthcare (Robins et al., 2005; Šabanović et al., 2013) or personal assistantship (Romero et al., 2017). However, much more work is needed to determine the extent to which humans can, and are willing to, perceive non-human agents as appropriate social interaction partners, as well as to determine a set of physical and behavioral features that could potentially induce such perception (see Wiese et al., 2017; for a review).

By default, humans are perceived as having “minds of their own” (Epley et al., 2007; Gray et al., 2007), which conveys an assortment of assumptions about their moral rights and responsibilities (see Waytz et al., 2010a; for a review). While anthropomorphism, or imbuing non-humans with human-like qualities, is a universal human tendency (see Epley et al., 2007; for a review), such perceptions are not binary but rather vary based on the observer, the observed agent and the environment. When a non-human agent is perceived to have high amounts of human-likeness, individuals adopt the “intentional stance” (Dennett, 1989), or the belief that the actions carried out by the agent are the result of “having a mind” that is capable of rational, intentional choices (Gray et al., 2007). The adoption of this belief has strong overall effects on our perceptions of these agents. The belief that behavior is intentional can affect the allocation of attentional resources and increase sensitivity to subtle social cues such as gaze direction (Wykowska et al., 2014; Caruana et al., 2017), which can help us communicate important social information effectively (Frischen et al., 2007; Mutlu et al., 2009). On the other hand, mindful agents are more likely to be perceived as deserving punishment for wrong-doing (Gray et al., 2007), unfair action from such agents are more likely to inspire disgust (Sanfey et al., 2003), and the perception that inflicted pain was intentional can increase the sensation of pain (Gray and Wegner, 2008).

The extent to which non-human agents can trigger the perception of mind is still an active topic of study in Human-Robot Interactions (HRI; Wiese et al., 2017; Iwasaki et al., 2019; Schellen and Wykowska, 2019), and important consideration in Human-AI Interactions (HAI). Some studies have demonstrated that only agents with very humanlike physical appearance can elicit humanlike social interactions (MacDorman and Ishiguro, 2006) or expectations of social experience (Martini et al., 2016). In real world interactions, it will be necessary for agents to maintain the appearance of “having a mind” over the course of dynamic social interactions by displaying socially plausible and adaptive behaviors. Behavioral triggers for mind perception include the action of engaging in eye contact with human interaction partners (Kompatsiari et al., 2019), making humanlike facial expressions (Breazeal and Scassellati, 1999), making mistakes (Salem et al., 2013), and presenting unpredictable or random behaviors (Short et al., 2010; Waytz et al., 2010b; Hayes et al., 2014). However, though unpredictable or random behaviors are often accompanied by a decrease in positive perceptions of the agent, presenting challenges if agents are designed for long-term relationships.

Some research has even demonstrated that brain areas involved in social-cognitive processing, such as the action-perception system, are similarly sensitive to actions performed by humans and mechanistic robots as long as the stimuli were non-repetitive actions and the motion produced by the robot was reproduceable by a biological organism (Gazzola et al., 2007; Bisio et al., 2014). Humans even ascribe intentions to videos of cartoons or even moving simple shapes (Heider and Simmel, 1944). However, others have shown that non-human social agents do not activate the higher-order social brain areas to the same extent that human interaction partners do (Sanfey et al., 2003; Takahashi et al., 2014; Wang and Quadflieg, 2015).

Many questions still remain as to how non-human social agents can be designed to trigger mind perception and the same level of activation in social brain areas, and these will require systematic and cross-disciplinary research (Wiese et al., 2017). A useful approach is to conduct research that investigates how distinct (or similar) AI performance is perceived compared to human performance. While not all AI are created in the same way, it is important to start asking these questions with state-of-the-art AI that is developed to produce humanlike behavior as a basis for understanding design criteria. Another benefit of this approach is that the development of AI that is inspired by cognitive and biological mechanisms of human learning and decision making can bridge the gap between the fields of AI and human-centered science for mutual benefit (Hadfield-Menell et al., 2016; Marblestone et al., 2016; Romero et al., 2017; Rabinowitz et al., 2018).

However, when the design of non-human agents reach the level of sophistication where they are so humanlike that they can actually deceive people into believing that they are human actors can pose societal hazards. We have seen recent evidence of the success of “bots” that pose as humans in order to disseminate misinformation across social media (Zaleski, 2016), or scam people on dating sites (Huhn, 2019). Publicly available algorithms can be used to make “deepfakes” that depict women in pornographic content without their consent (Wagner and Blewer, 2019) and could easily be used to create false evidence to promote political goals (Schwartz, 2018). Considering these applications, it is important to examine how sensitive people are to true humanness when they have no explicit information about a social actor's identity.

The study of convincingly humanlike AI can draw inspiration from history. Alan Turing's test of machine intelligence (now commonly called the Turing test) postulated that a machine should be considered intelligent if it could convince at least 30% of human evaluators that it was a human after engaging in 5 min of unrestricted conversation (Turing, 1950). While the evaluation was meant to be on the basis of the natural language conversation through text, AI designers discovered that adding certain behaviors (like including typos, delaying the response to emulate human reaction time, and intentionally not answering questions) increased the likelihood that the algorithm would be rated as a human (Epstein et al., 2009). In 2014, the first chatbot passed the Turing test, though it has been pointed out that clever use of a back story (giving the agent the identity of a 13 year old Ukrainian boy to excuse grammatical issues or a lack of knowledge) and outside these behavioral features were used to cheat the test (Warwick and Shah, 2016).

While the original Turing test was conducted on the basis of a typed conversation, some behavioral Turing tests have been published in recent years (Pfeiffer et al., 2011; Osawa et al., 2012; Wykowska et al., 2015; Tulk et al., 2018) in which humans judges must distinguish between human and AI actors strictly on the basis of observed behaviors. There are several important takeaways from this research. One is that it is more difficult to distinguish between humans and AI on the basis of behavior alone (Osawa et al., 2012; Tulk et al., 2018). People may have presumptions about what robot vs. human movement looks like (e.g., quick onset of motion) that affect judgments of humanness (Wykowska et al., 2015). Also, evaluations of how humans are likely to behave in specific interaction contexts (e.g., cooperative vs. competitive) are used to judge humanness, and the assumptions of humanlike behavior are different depending on this context (Pfeiffer et al., 2011).

An important aspect to consider for behavioral Turing tests is that peoples' perceptions of agents are affected by subtle cues such as timing of a response or movement (Epstein et al., 2009; Wykowska et al., 2015) or the way an agent communicates (Short et al., 2010). Videogames provide the perfect environment to study how humans interact with AI because they are already developed for rich social interactions, and many provide the opportunity for researchers to build systems to capture behavioral data from within the game through custom modifications. Videogames have been used both as a platform for training and evaluating AI (Laird and VanLent, 2001; Mnih et al., 2015; Bard et al., 2020), and as a way to investigate how human and AI performances are perceived by measuring human behavior and subjective experience (Ehsan et al., 2018; Tulk et al., 2018) as well as physiological measures (Lim and Reeves, 2009).

In order to begin to understand how to create social AI that behaves and is perceived as an appropriate social interaction partner, this research attempts to answer the following questions: (1) How well can people distinguish between human and AI performance on the basis of behavior within a complex environment, and (2) how do social interactions and perceptions of interaction partners differ when an AI is developed with the capacity to think and act socially?

This study attempts to answer these questions by first observing how humans develop opinions and relationships with human and AI co-players within the complex, naturalistic multiplayer videogame. The game is *Don't Starve Together* (Klei Entertainment, 2016). A research modification has been created to provide the environment for a behavioral Turing test and collect data related to game behaviors and interactions with different co-players. Secondly, a “social” AI has been developed to have “a mind of its own” and uses humanlike motivations to play the game and interact with human players. The cognitively plausible AI system learns from social interactions with other players in order to determine its own interaction strategy and understanding of the social context of interactions.

There are two hypotheses for this experiment: (H1) Even within a complex environment, participants will be able to distinguish between the behaviors of human players and “simplistic” AI co-players that simply emulates human behaviors with no overarching motivations. This finding would be consistent with Wykowska et al. (2015). While our pilot study (Tulk et al., 2018) did show that it was difficult to differentiate between a human and a “simplistic” AI co-player, the human was instructed to play in a manner that was similar to how the AI was programmed, and the interaction was relatively brief (15 min), which likely affected participants' ability to judge. (H2) It will be more difficult for participants to distinguish between the behaviors of human players and “social” AI co-players that have been designed to play the game and interact with humans on the basis of cognitively plausible, humanlike motivations. This finding would be in line with Osawa et al. (2012), which demonstrated that it was more challenging to differentiate between human and AI behavior when the AI was developed to emulate human-human communication qualities. Additionally, behavioral measures and qualitative data was collected in order to explore the range of behaviors and cues that affected overall perceptions of these co-players.

## Methods and Materials

### Participants

A total of 83 undergraduate students (mean age = 20.3, SD = 2.60; 43 females) participated in this study. Participants were compensated for their participation with credits through the SONA psychological research system, which could be applied for course credit in psychological classes offered at George Mason University. Seventeen participants were removed due to technical difficulties or glitches associated with the modifications made to the game (e.g., game crashing after the experiment was started, the modification not being started properly, internet connectivity issues, agent continuing to run into a wall or standing still for more than half the experiment) or incomplete datasets. This removal resulted in a total of 66 usable datasets (mean age = 20.6, SD = 2.81; 37 female). The only screening criterion was that participants had no prior experience playing the game. Participants reported spending an average of 5.8 h (SD = 2.93) on a computer per day and an average of 4.7 h (SD = 8.09) playing videogames per week.

For this experiment, participants engaged in 30 min of unrestricted, non-verbal game-play (i.e., no text or audio communication) with a human or AI co-player. The humans' co-player was a second *participant* (human: 22 participants; mean age = 21.3, SD = 2.34; 13 female) who participated in the study at the same time and simultaneously judged one another while playing according to their own preferences. Two AI co-players were used in this study: a *simple AI* (22 participants; mean age = 20.3, SD = 3.12; 9 females) that used a behavior tree to play in a manner that emulated humanlike actions in the game, and a *social AI* (22 participants; mean age = 20.2, SD = 2.94; 15 females) that was designed to perceive and learn from participants, and played the game based on plausible cognitive mechanisms for survival and social interactions. Participants were randomly assigned to their experimental group prior to arriving to participate in the study, however, most of the data collection for the social AI occurred a few months after data collection finished for the other two experimental groups, as it took longer to create a stable version of the social AI. Additionally, since the tandem human condition required two participants to be present at the same time, if one participant did not show up, the other participant was re-assigned to either the social or simple AI group. This research was conducted with approval from the university's ethics committee (i.e., the institutional review board) and was carried out ethically. In total, each participant took an hour to complete to protocol.

### Multiplayer Videogame

*Don't Starve Together* is an immersive, multiplayer wilderness survival game where players collect resources (e.g., food and fire wood) and craft tools and other objects (e.g., hats, armor, hand tools) to trade or use to survive. Players can choose to act however they desire within the game, including exploring a vast environment, fighting or befriending creatures they encounter, or building elaborate tools to help them survive and progress in the game.

Two players can choose to interact with one another in a variety of ways, including following one another around the map, exchanging goods (e.g., food, clothing, tools), fighting one another, and assisting each other in hunting food or fighting monsters. Because the two players can see one another's avatar in the top-down vision of the world, and see each player's icon on the map, each can be observed in relation to their low level behaviors (e.g., moving around the environment or making micro adjustments to avoid obstacles or interact with the environment, how frequently and how long they pause to look at their menu or at items in the environment, how quickly they react if an interaction is engaged by a player or an entity in the environment) as well as higher level behaviors (e.g., if and when they cook food, consume food or craft tools, how often they go to home base, if they assist the participant by helping to hunt or fight entities, or give gifts, or if they act competitively by snatching up all the valuable resources or intentionally attacking the other player, and how they respond based on the social actions of the other player). Overall, many aspects of behavior can be construed as social cues, including direct interactions (e.g., giving gifts, attacking, or helping a player hunt or fight other entities), or less direct interactions, such as how close co-players stayed to the participant, if co-players followed participants, or appeared to watch the participant as they played the game.

**Figure 1:**
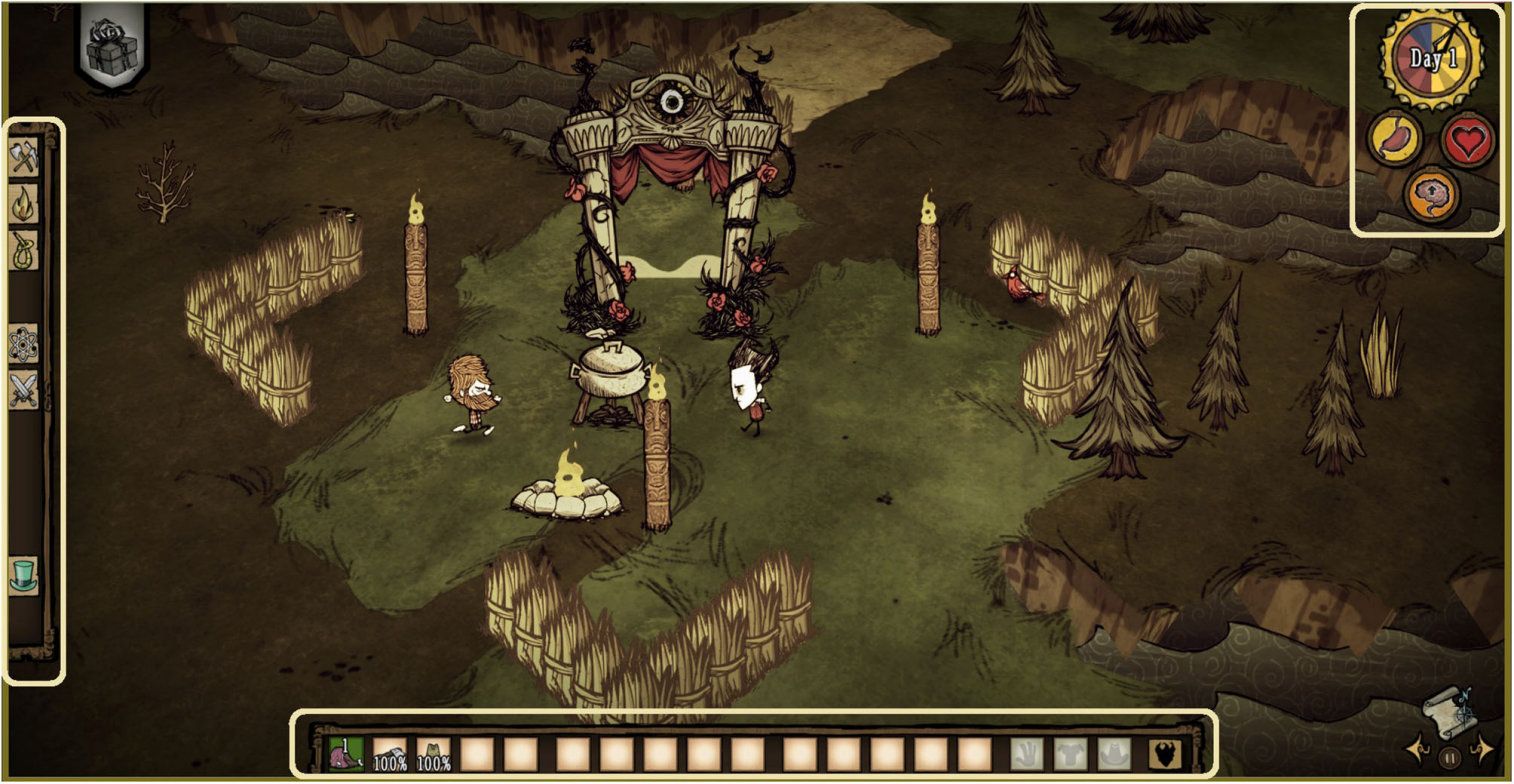
A Typical Game Scene: Both players (Wilson and Woodie) are in view. Both players are within the “home base” that featured some barriers, a cook pot and a constant light source that offered protection at night. The game statistics (hunger, health, sanity) are displayed in the upper right corner of the screen. The player's inventory is displayed on the bottom of the screen. On the left side of the screen is the “crafting tab” where players can view and pick from different.

**Figure 2:**
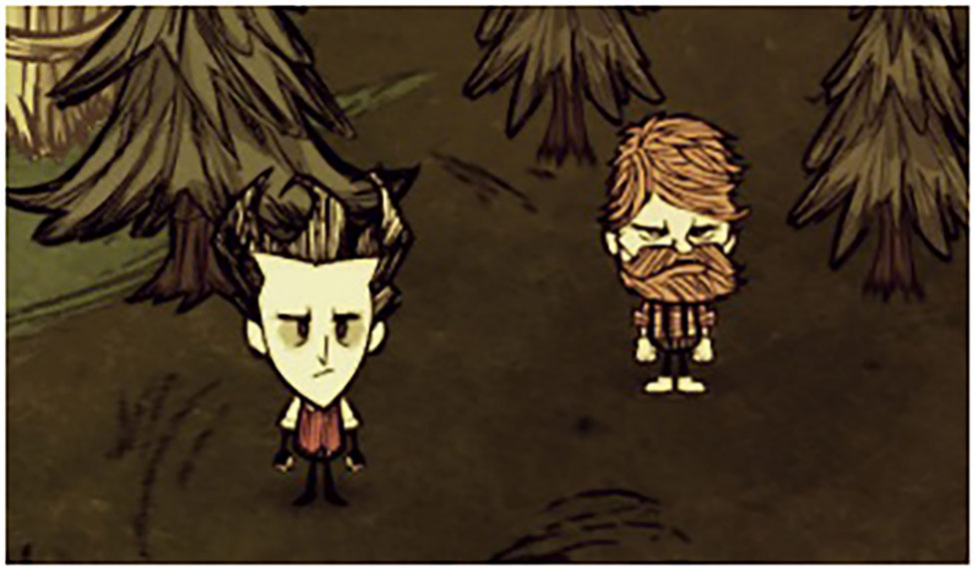
Player avatars: Wilson (left) was played by the participant; Woodie (right) was played by half of the participants in the human condition and by the confederate human, simple AI, and social AI.

The game operates on a day cycle, with a clock at the top right side of the screen indicating when it is morning, evening or night. At night, the entire field turns dark, and players must find light to see their environment and stay alive. Players can track how well they are performing by looking at their health, hunger, and sanity levels that are displayed on three icons in the top right of the screen, just under the clock. Players can also see how many items they have stored in their inventory (displayed at the bottom of the screen) and can interact with the crafting tab displayed on the left side of the screen to determine what items they can build given the resources carried in their inventory; see Figure 1. A modification to the game code was created for this research, such that player metrics and behaviors were recorded. Additionally, a “home base” was added with a constant light source such that participants and co-players could always return home at night for safety. Home base also offered a natural location where players might encounter each other over the course of the experiment. The game features a map to help players navigate the environment, and icons for each player were placed within the map so that players could always find each other. AI agents were also created for this study by modifying and further developing existing game AI and by using an existing playable avatar (i.e., “Woodie,” see Figure 2).

### Co-players

Participants interacted with co-players (human or AI) within the game for 30 min. The chat function was disabled such that all interactions were behavioral. The two avatars that players and co-players used were “Wilson” and “Woodie”; see Figure 2. These two avatars were selected for their similar features and relatively normal appearances compared to other available avatars in the game. In the AI conditions, the participant played as Wilson and the AI co-player played as Woodie. The avatar assignment was specified to make the start up procedure as straightforward as possible for research assistants, as improperly starting the game with the research modifications or improperly adding the co-player to the game could cause the game to crash or result in missing data.

#### Human Co-players

In the human condition, two participants were recruited to participate at the same time and unwittingly played the game together, such that each participant was also the co-player of another participant. Human co-players therefore had no prior experience with the game and could play the game and interact however they wanted. All human players (participants/co-players) were aware that they would be asked whether they thought their co-player was a human or AI at the end of the experiment. However, participants were given no explicit instructions on whether and how to evaluate co-players. In this way, participants and co-players should have behaved in similar “humanlike” fashion over the course of the game. The player avatars (Wilson and Woodie) were arbitrarily assigned to participants.

#### Simple AI Co-players

**Figure 3:**
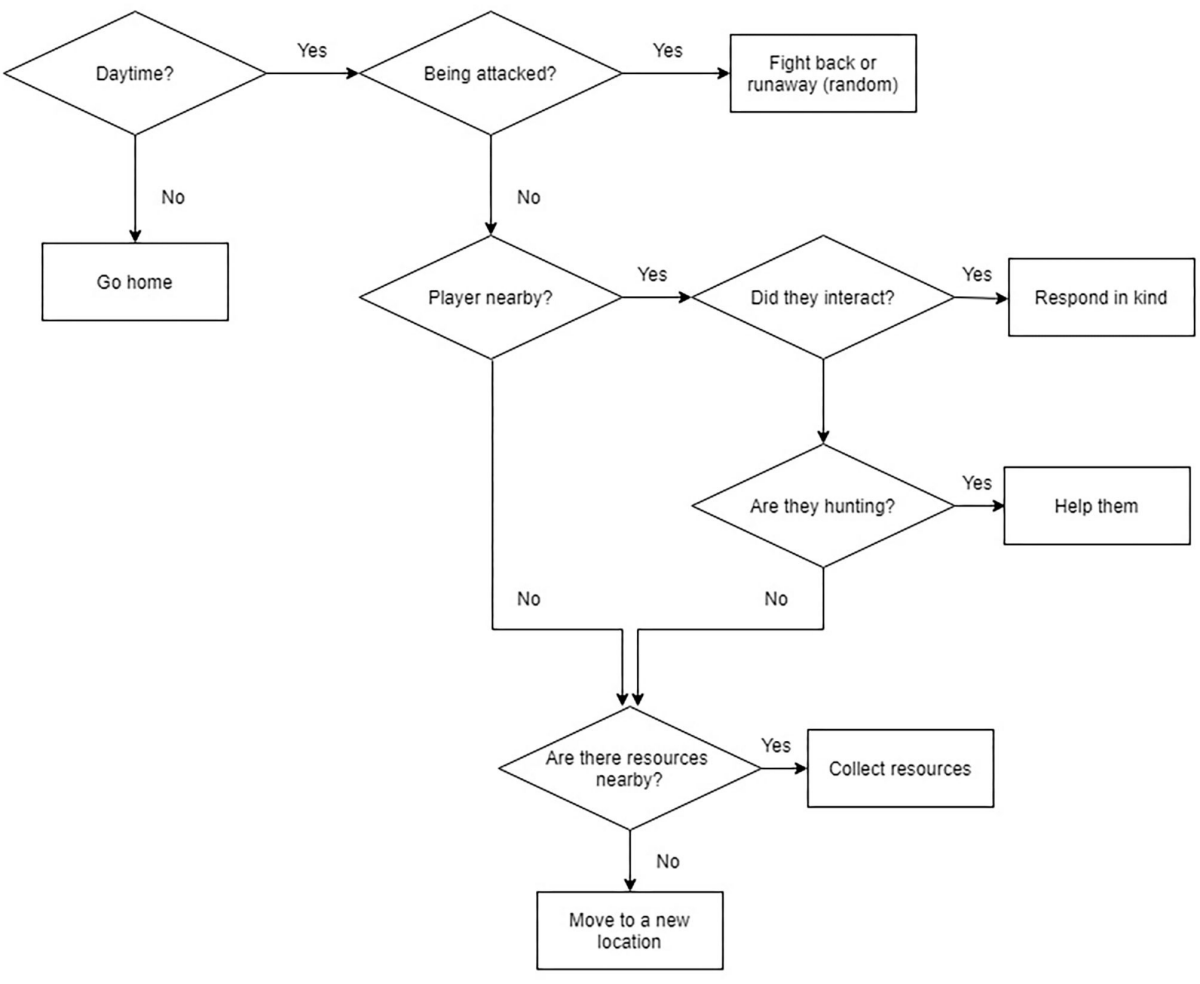
The Simple AI's behavior tree. The simple AI's behavior was governed by this behavior tree.

A simple AI agent was created for a pilot study (Tulk et al., 2018) and was developed further based on pilot-participant feedback. This agent's behavior was governed by a behavior tree (i.e., no learning algorithm or learning involved) that was intended to react to game stimuli in a way that emulated humanlike behaviors within the game, but the agent had no motivations for making decisions about how to behave in the game; see Figure 3. In “social interactions,” the agent was programmed to act with reciprocity (i.e., if attacked, it would attack back; if given a gift, it would give a gift back within a few minutes), but would never initiate these interactions on its own. Since the timing for returning gifts was random, it was possible that the co-player had difficulty catching up with participants within the expansive environment to be able to return gifts. Additionally, this AI was only sensitive to very salient interactions (i.e., being attacked by participants, being handed a gift, or having a gift dropped very near to its avatar in the game) and would not notice actions such as participants chasing it or running into it. It could, however, notice when a participant was fighting another entity in the game if they were very near-by, and would assist participants in fighting.

#### Social AI Co-players

**Figure 4:**
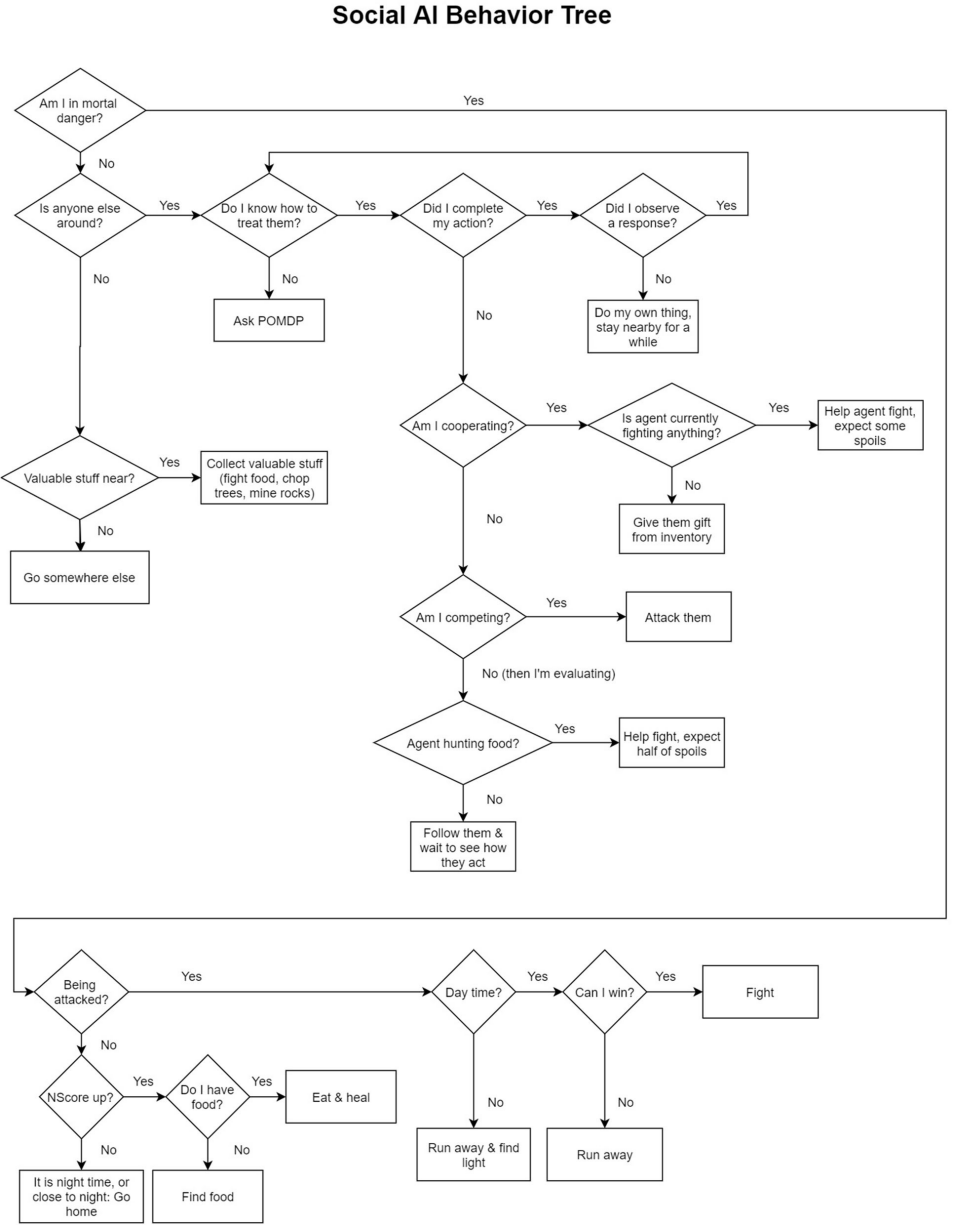
The Social AI's Behavior Tree. The social AI's behavior consisted of two components. The first component was a behavior tree that was designed from participant's statements about what constituted humanlike behavior in the game. The social AI kept track of its own “neediness” (NScore) based on its current player stats (health, hunger, and sanity) and how many resources it had in inventory, or how well it was currently surviving in the game and made decisions based on how needy it was in the moment. The second component involved a POMDP that kept a memory of other agents it interacted with, estimated for itself the social context, and made decisions based on how it was being treated by the participant.

As an important aspect of HAI involves the way in which AI agents perceive and decide to interact with humans, a socially and cognitively plausible AI agent was created to decide for itself how to play the game and interact with participants. The model consisted of two independent components. The first component was a new behavior tree that determined all survival-based behaviors and was designed from participants' descriptions of what constituted “humanlike” survival behaviors and motivations (analysis not presented in this paper). In contrast with the simple AI, this behavior tree was designed to emulate humanlike motivations when playing the game, such as making decisions to aid in survival and interacting with human players based on its current survival state and the perceived social context. The agent kept track of its own health, hunger and stock of its inventory (combined into one measure called “neediness score” or “NScore”) in order to make decisions that would increase its chances of survival. As an example, it only collected items that were valuable and which it did not already have an ample supply of, hunting easy prey for food and deciding whether or not to engage in fights with other creatures in the game that could fight back; see Figure 4.

The second component was a Partially Observable Markov Decision Process (POMDP) used to represent the social context and determine how to treat participants (namely, to act cooperatively, competitively or tentatively) based on its own perception of the human's actions (cooperative or competitive). POMDPs have been used to dictate AI social behavior (Rabinowitz et al., 2018) and are particularly suited for representing environments in which an agent does not have perfect knowledge about the current state or what to expect. The POMDP was adapted from the open source POMDPy (Emami et al., 2015), and made decisions via a Monte-Carlo Tree Search algorithm that simulated the agent's own choices (Silver and Veness, 2010) and the choices of social partners to determine how to interact with others to achieve the best outcome given its current representation of the social context. The social AI represented every action and observation as a two-person matrix game with turn taking, where each player can decide to act either *cooperatively* or *competitively* (four possible outcomes), plus an additional option for the social AI to wait and *evaluate* the other player rather than act, resulting in six possible outcomes. The social AI was designed to use the values associated with each outcome (i.e., a payoff matrix describing relative rewards and punishments for joint outcomes) to determine how to interact within the game in order to maximize its expected reward, but also to update the payoff matrix based on what it observed from a co-player (i.e., the participant). This meant that not only did it develop a preference for how to interact, but also decided how to interact based on what it believed the other player would do within a social context (e.g., choosing to cooperate if it believed this act would be met with cooperation). The payoff matrix was initially configured to assume a context favoring mutual cooperation. As the social AI interacts with the participant, the payoff matrix is updated and may shift toward different types of games. In this way, the agent is able to change its strategy for interacting with participants.

### Apparatus

Two copies of the game *Don't Starve Together* were purchased and modified to record participants' in-game behavior and interactions with co-players. Modifications were also made to make the game a little easier for the participant (e.g., they could never actually die, but were not informed of this fact), and the chat function was disabled to ensure that all interactions were behavioral. The game was played on PCs through the Steam gaming platform (Valve, 2003). Participants were given the option to use either an Xbox style controller or mouse and keyboard. All questionnaires were administered through Google Forms. Interviews were conducted verbally and transcribed by the researcher.

### Measures

#### In-game Behavioral Measures

While playing the game, various measures associated with the participants' in-game behaviors, performance, and interactions with co-players were recorded. They included: (1) distance between the participant's and co-player's avatars within the game environment (measured in approximate centimeters on the monitors), (2) how often participants engaged in interactions such as giving/receiving items to/from co-players, how often participants attacked co-players/were attacked by co-players, and how often they engaged in joint hunting (e.g., rabbits) or fights with other game entities that could fight back (e.g., giant spiders). Data was recorded automatically from the game once any actions of interest occurred and at various times throughout game play, resulting in approximately 400–1,000 records for each participant. Distance was recorded approximately once every 5 s. Importantly, while a “give” function was included such that participants could directly hand gifts to the co-player and would receive a notification when a co-player gave them items with the same function, many participants indicated that they would drop items near the co-player's avatar instead. Both AI co-players considered this to be a gift giving action, but it cannot be said with certainty that participants (who acted as human co-players for other participants) would have noticed this as it is significantly less salient, therefore estimations of gifts received from human co-players only included gifts given directly through the “give” function.

#### Surveys

Prior to playing the game, participants filled out a generic demographics survey. After playing the game, participants reported the extent to which they trusted the co-player and the extent to which they felt like the game and co-player were “real” (Schneider et al., 2004). Both qualities were reported on Likert measures out of 10 points.

#### Turing Test and Interviews

After playing the game and responding to survey questions, participants were interviewed on what they thought the other player's identity was (i.e., the Turing test) and what cues led them to this judgment. Additional data was collected related to overall perceptions of agents and the perceived social context of the interaction, but these results will not be discussed in this paper as a much more thorough qualitative analysis is planned.

### Procedure

At the beginning of the experiment, participants read the consent form and confirmed that they consented to be in the study, then filled out the demographic questionnaire and were given instructions for the experiment. Participants were then told that they would be playing *Don't Starve Together* with another player, and were instructed that they could do whatever they wanted in the game and toward the co-player, and that at the end we would ask them if they believed that the co-player was a human or an AI agent. Researchers randomly assigned participants to these experimental groups (i.e., participating with a tandem human, simple AI, or social AI) prior to their arrival, and participants were not made aware of this assignment. They were also informed that chat within the game was disabled, and that all communication within the game would be behavioral.

Participants practiced playing the game for 5 min where they were given tips on how to play the game and were allowed to ask questions. Participants were then asked to leave the computer area while the experimenter brought the co-player into the game by initializing their avatar in the home base. In the human condition, an online server was created on one lab computer where Wilson was selected as the participant's avatar, and researchers connected the secondary computer to the server and initialized as Woodie, who was played by another participant in another room. Lab rooms were on separate floors of the same building so that participants in the human condition did not encounter one another, which could have potentially biased the experiment. In both AI conditions, a local server was created with Wilson as the participant's avatar, and a new instance of the AI co-player (simple AI or social AI) was created with Woodie as the avatar. In the social AI condition, the POMDP was running in a terminal in the background, but was not visible to the participant at any point. Once both players were initialized within the environment, the participants played the game with their co-player for approximately 30 min. After playing for 30 min, the game was turned off and participants responded to survey questions. Finally, participants were verbally interviewed about the perceived identity of the co-player and what behavioral cues led to this identification. At the end of the experiment, participants were told the actual identity of their co-player and thanked for their participation.

### Analysis

Performance on the Turing test was evaluated by comparing the frequency that all co-players were rated as humans in a forced choice response. Additionally, the accuracy of the binary responses of humanness were compared against chance (similar to Wykowska et al., 2015) to examine how sensitive participants were to humanness (or the absence of it) when compared to the performance of simple and social AI co-players.

In order to compare how participants and co-players engaged in interactions and how symmetrical these interactions were (i.e., if participants and co-players reciprocated interactions engaged by one another or if one player tried to engage the other more frequently), the scored difference in interactions engaged by participants vs. co-players was calculated by the following equation using data recorded from in-game behaviors:

$$\begin{array}{l} \text{Scored Difference}=\frac{\left(\text{p}-\text{c}\right)}{\left[\frac{\left(\text{p}+\text{c}\right)}{2}\right]} \end{array}$$

Where p = total interactions engaged by participants,

c = total interactions engaged by co-players

Comparing percent difference is a common approach to compare two experimental values in a standardized way (Glen, 2016) and offers the opportunity to compare the extent to which interactions within the game were reciprocated by participants and co-players. Here, a variation of the percent difference equation is used as a non-absolute value such that positive and negative values of the scored difference can be evaluated, where greater positive scores are associated with relationships where participants engaged in interactions that were unreciprocated by co-players, and greater negative scores with relationships where co-players engaged in interactions that were unreciprocated by participants. The motivation for using this measure is the acknowledgment that reciprocity is a strong expectation in human-human social interactions (Gouldner, 1960; Axelrod and Hamilton, 1981).

Analysis of Variance was used to examine the relationship between co-player identity and perceived humanness on participants' in-game behaviors (i.e., average distance between players and the scored difference in participant-engaged vs. co-player-engaged interactions) as well as explicit survey ratings of how much participants trusted co-players and perceived them as a “real person.” All reported *post-hoc* analyses were conducted with Tukey's HSD (Abdi and Williams, 2010).

In order to examine the behavioral cues participants described when asked how they made determinations of humanness, participants' natural language responses were analyzed and coded by two raters. Cohen's Kappa was calculated to determine the inter-rater reliability, and χ^2^ tests were used to investigate the relationship between perceived humanness, co-player identity and the most frequently occurring cues.

## Results

### Perceived Humanness and Sensitivity to Human Behavior

Overall, 10 out of 22 participants (45%) perceived human co-players as humans, five out of 22 (23%) perceived the simple AI co-players as humans, and seven out of 22 (32%) perceived the social AI co-players as humans. The overall accuracy of participants who played with human and simple AI co-players was 61%, which is a level of performance that is not significantly above chance, *t*(43) = 1.53, *p* = 0.067; *d* = 0.242, one tailed. The overall accuracy of participants who played with human and social AI co-players was 57%, with a performance level that was not significantly greater than chance, *t*(43) = 0.90, *p* = 0.186; *d* = 0.116, one-tailed.

### In-game Behaviors

**Figure 5:**
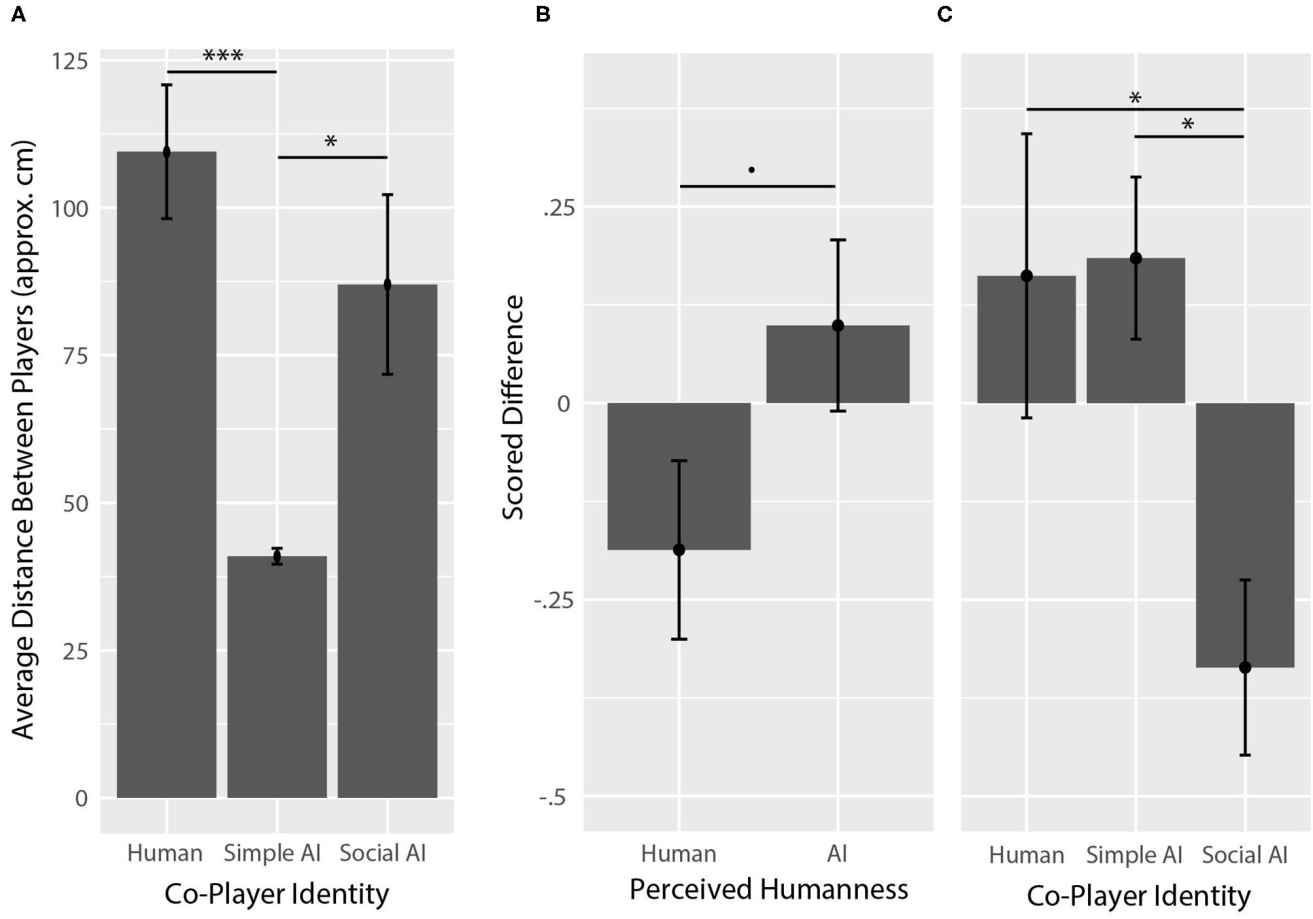
In-game behaviors. **(A)** In-game distance between participant and co-player. The distance between participants and the simple AI co-player was significantly smaller than the distance between participants and human co-players, and participants and social AI co-players. **(B)** Scored difference between in-game social interactions engaged by participants vs. co-players, across perceived humanness. Co-players that were perceived as human engaged participants more often on average than they were engaged by humans, though the difference was only approaching significant. **(C)** Scored difference between in-game social interactions by co-player identity. Social AI co-players engaged participants more often than they were engaged by participants on average. Asterisks (*) correspond to levels of statistical significance, where one asterisk means *p* values were less than 0.05, two asterisks means *p* values were less than 0.01, and three asterisks means *p* values were less than 0.001.

The average distance between participants and co-players within the game can be seen in Figure 5A. Analysis of variance showed that average distance was significantly different across co-player identity (*F*_(2,60)_ = 9.84, *p* < 0.001, η_*p*_^2^ = 0.223), but not across perceived humanness (*F*_(1,60)_ = 0.99, *p* = 0.324, η_*p*_^2^ = 0.016). A *post-hoc* analysis (with Tukey's HSD) showed that the average distance between participants and simple AI co-players (M = 40.9 approx. cm, SD = 6.3) was significantly less than the average distance between participants and human co-players (M = 109.5, SD = 53.2; *p* < 0.001, *g* = 1.77) as well as between participants and social AI co-players (M = 87.0, SD = 71.4; *p* = 0.012, *g* = 0.89), but the average distances between participants and human co-players compared to social AI co-players was not significant (*p* = 0.323, *g* = 0.36).

Average scored difference in participant-engaged vs. co-player-engaged interactions with respect to perceived humanness and co-player identity can be seen in Figures 5B,C. Analysis of variance showed that scored difference in participant-engaged vs. co-player-engaged interactions was significantly different across co-player identity (*F*_(2,60)_ = 4.79, *p* = 0.012, η_*p*_^2^ = 0.141), but not significant across perceived humanness (*F*_(1,60)_ = 3.30, *p* = 0.074, η_*p*_^2^ = 0.052), where co-players that were perceived to be humans had a negative average score (M = −9.3%, SD = 26.6%), indicating that co-players that were perceived as humans engaged with participants more often than they were engaged with by participants overall. A *post-hoc* analysis showed that the scored difference associated with participant vs. social AI co-player engaged interactions (M = −33.6%, SD = 52.3%) was significantly lower than the scored difference associated with participant vs. human co-player engaged interactions (M = 16.2%, SD = 84.8%; *p* = 0.032, *g* = 0.69) and participant vs. simple AI co-player engaged interactions (M = 18.5%, SD = 48.4%; *p* = 0.024, *g* = 1.02).

### Survey Responses

**Figure 6:**
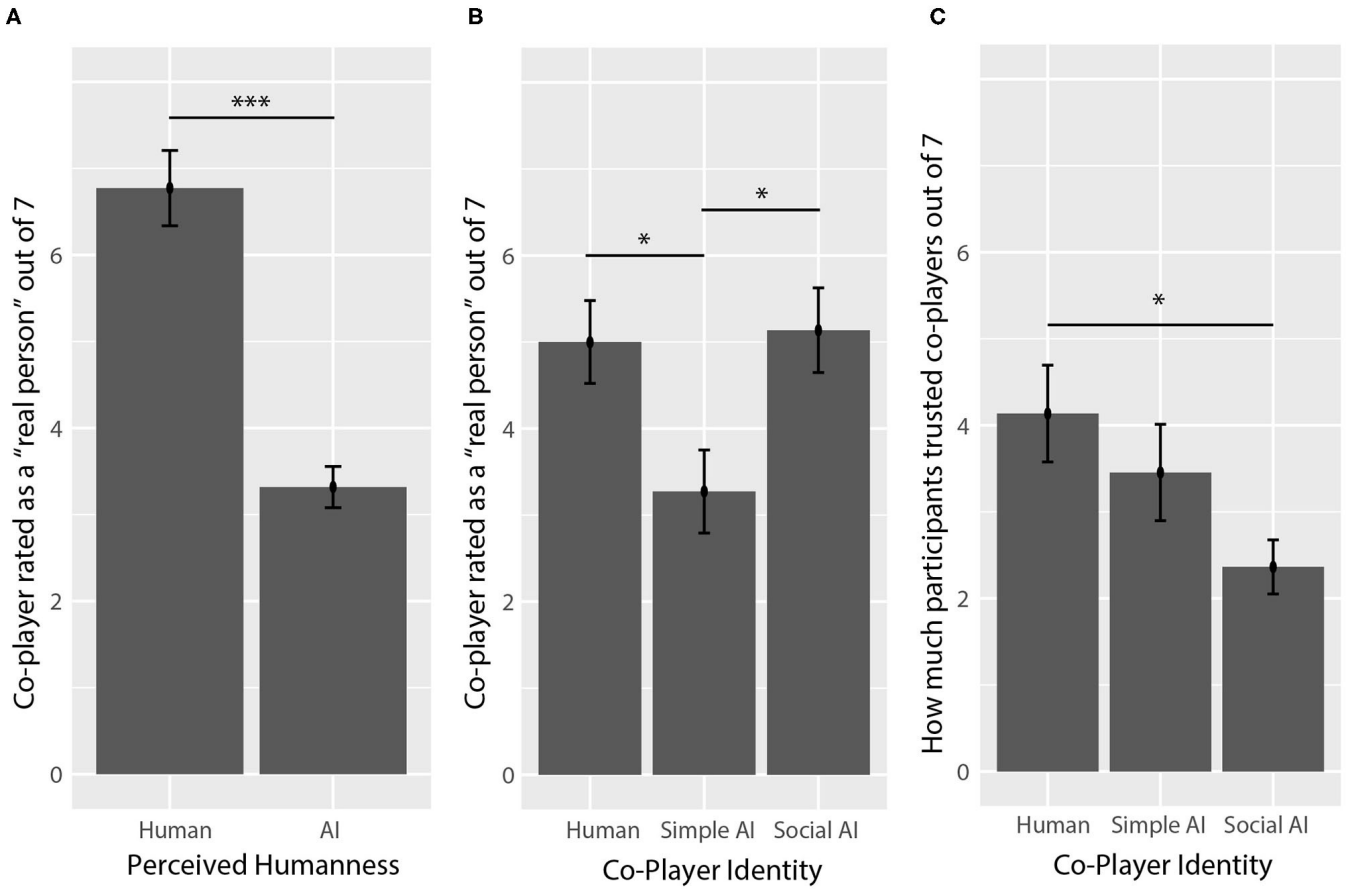
Explicit ratings. **(A)** Co-players that were perceived as humans were perceived as being a “real person” more than co-players perceived as AI. **(B)** Human co-players and social AI co-players were perceived as a “real person” more than simple AI co-players. **(C)** Participants reported that they trusted social AI co-players less than the human co-players. Asterisks (*) correspond to levels of statistical significance, where one asterisk means *p* values were less than 0.05, two asterisks means *p* values were less than 0.01, and three asterisks means *p* values were less than 0.001.

Average ratings of how much participants trusted co-players can be seen in Figure 6A. Participants reported different levels of trust depending on co-player identity (*F*_(2,60)_ = 3.273, *p* = 0.045, η_*p*_^2^ = 0.101), but not perceived humanness (*F*_(1,60)_ = 0.23, *p* =0.633, η_*p*_^2^ = 0.004). A *post-hoc* analysis showed that participants trusted social AI co-players (M = 2.4/7, SD = 1.5) significantly less than human co-players (M = 4.1/7, SD = 2.6; *p* = 0.034, *g* = 0.81).

Average ratings of how much participants perceived their co-player as a “real person” is shown in Figures 6B,C. Participants' ratings were impacted by co-player identity (*F*_(2,60)_ = 8.66, *p* < 0.001, η_*p*_^2^ = 0.141), and perceived humanness (*F*_(1,60)_ = 56.41, *p* < 0.001, η_*p*_^2^ = 0.485), where co-players perceived as humans received higher ratings (M = 6.8/7, SD = 2.0) than co-players perceived as AI (M = 3.3, SD = 1.6). A *post-hoc* analysis showed that participants perceived simple AI co-players (M = 3.3/7, SD = 2.3) as a “real person” significantly less than human co-players (M = 5.0/7, SD = 2.2, *p* = 0.037, *g* = 0.71) and social AI co-players (M = 5.1, SD = 2.3, *p* = 0.022, *g* = 0.81), who actually received the highest mean ratings, while the difference between the human and social AI co-players was not significant (*p* = 0.978, *g* = 0.10).

### Behavioral Cues of Humanness

The natural language responses participants provided to indicate what behavioral cues helped them determine humanness were coded independently by two separate raters. A summary of these cues is presented in Table 1, including the top five frequently occurring cues, Cohen's Kappa for inter-rater reliability, the relative frequency for codes occurring across perceived humanness and co-player identity, and results from χ^2^ tests.

**Table 1:** Cues participants used to make determinations of humanness.

**Cue**	**Total count**	**Cohen's Kappa**	**χ^2^ between raters**	**Relative frequency of cue by perceived humanness**		**χ^2^ for perceived humanness**	**Relative frequency of cue by co-player Identity**		**χ^2^ for co-player identity**
**Not enough social interactions**	22	0.893	(1,65) = 48.38, *p* < 0.001	AI	41%	(1,65) = 2.14, *p* = 0.144	Simple AI co-players	45%	(1,65) = 0.51, *p* = 0.775
“*When the game started, they immediately walked away. I would expect that a person would try and tell if I was hostile or friendly.”*				Human	19%		Human co-players	33%	
							Social AI co-players	23%	
**Movement**	14	0.737	(1,65) = 31.21, *p* < 0.001	AI	27%	(1,65) = 1.70, *p* = 0.192	Simple AI co-players	36%	(1,65) = 4.33, *p* = 0.115
“*They were very active and their movement was fluid”*				Human	10%		Human co-players	14%	
							Social AI co-players	14%	
**Co-players** ***did*** **interact**	12	0.692	(1,65) = 25.92, *p* < 0.001	Human	43%	(1,65) = 9.99, *p* = 0.002**	Human co-players	24%	(1,65) = 0.74, *p* = 0.691
“*helped me chase the rabbit and helped with other tasks”*				AI	7%		Social AI co-players	18%	
							Simple AI co-players	14%	
**Not enough interactions with the environment**	12	0.614	(1,65) = 19.96, *p* < 0.001	AI	25%	(1,65) = 2.64, *p* = 0.104	Social AI co-players	23%	(1,65) = 0.51, *p* = 0.775
“*It didn't collect the logs after the trees were cut down”*				Human	5%		Simple AI co-players	18%	
							Human co-players	14%	
**Random or unpredictable behavior**	11	0.663	(1,65) = 24.06, *p* < 0.001	AI	14%	(1,65) = 0.00, *p* = 0.970	Social AI co-players	32%	(1,65) = 14.30, *p* < 0.001***
“*Player 2 would attack for no reason”*				Human	5%		Human co-players	0%	
							Simple AI co-players	0%	

Asterisks (*) correspond to levels of statistical significance, where one asterisk means p values were less than 0.05, two asterisks means p values were less than 0.01, and three asterisks means p values were less than 0.001.

**Figure 7:**
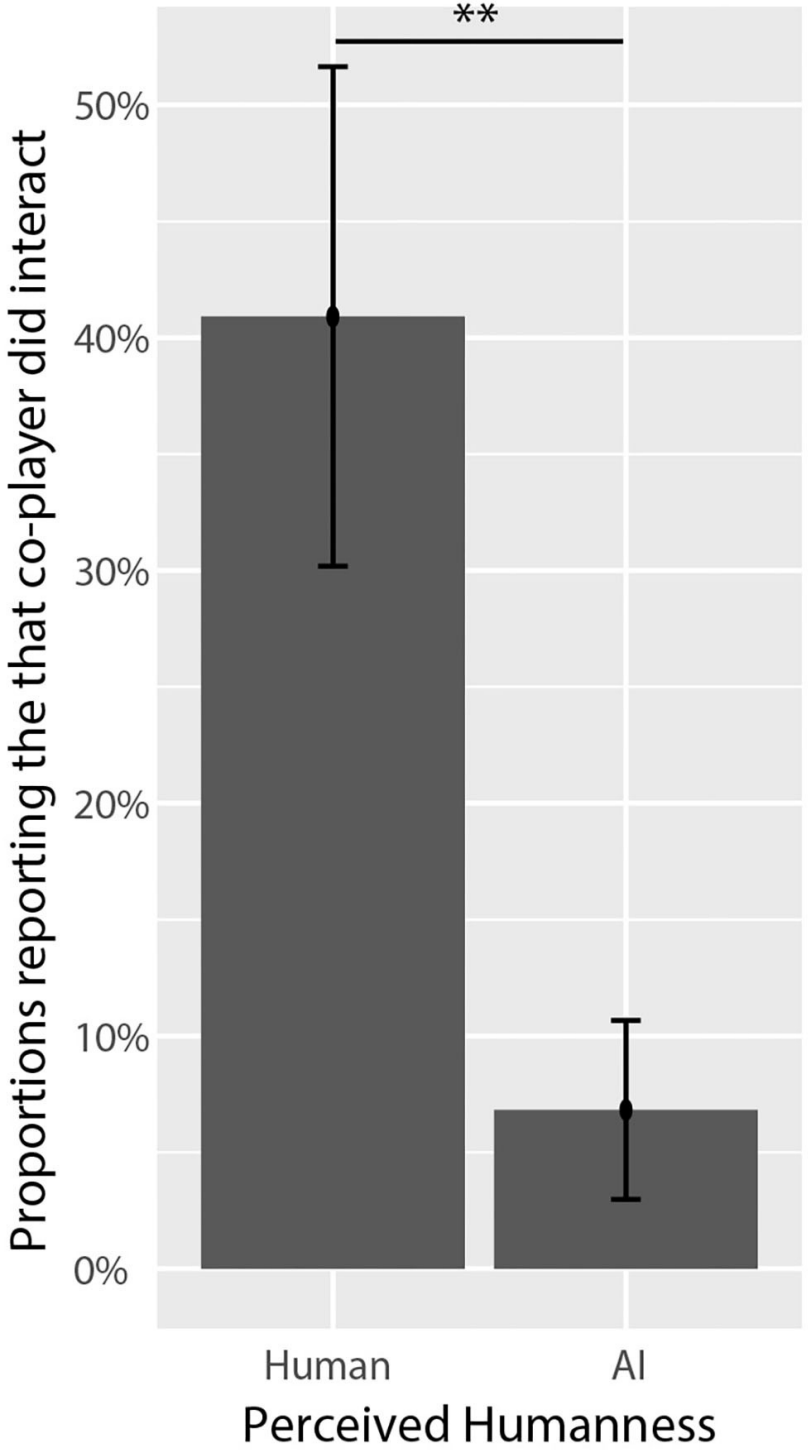
Proportions of participants who reported that their co-player interacted with them by perceived humanness. The cue that co-players did interact was significantly associated with the perception of humanness. Asterisks (*) correspond to levels of statistical significance, where one asterisk means *p* values were less than 0.05, two asterisks means *p* values were less than 0.01, and three asterisks means *p* values were less than 0.001.

The most frequently occurring cue used by participants when making determinations of humanness was the observation that the co-player did not engage in enough interactions with the participant. While this cue was more frequently associated with agents perceived as AI, the association was not significantly different with respect to perceived humanness or co-player identity. However, the cue that the co-player *did* interact with participants was more strongly associated with co-players perceived as humans; see Figure 7. With respect to co-player identity, the only cue (within the top 5) that differed significantly was the perception that the co-player acted randomly or unpredictably, which was only associated with social AI co-players.

## Discussion

Our study attempted to shed light on how sensitive humans are to complex behaviors of human and AI co-players within a naturalistic game environment. We compared participants' accuracy in distinguishing between the behaviors of human co-players and those of AI co-players that were “simplistic” (i.e., lacking any motivation for social interactions and the ability to perceive social context), or “social,” with a built-in capacity to sense social cues and determine for themselves how to interact with participants using cognitively plausible, humanlike motivations.

Our first hypothesis was that participants would be sensitive to performances by humans and be able to distinguish between human and simple AI co-players. The results from our behavioral Turing test were that participants labeled human co-players as humans more often than simple AI co-players, though the accuracy above chance was only approaching significant. However, participants reported that they perceived the simple AI co-player as a “real person” significantly less than human and social AI co-players, suggesting some support for rejecting the null hypothesis that humans are incapable of distinguishing between humans and simple AI co-players. This is in line with prior research in behavioral Turing tests has suggested that it can be challenging for participants to distinguish between human and AI players (Osawa et al., 2012; Tulk et al., 2018), but that humans may still have some sensitivity (Wykowska et al., 2015). It is also worth mentioning that human players were rated as humans <50% of the time, suggesting that participants were more likely to rate co-players as AI regardless of actual humanness. Since this Turing Test was conducted only based on behaviors, the lack of verbal communication may have made co-players seem less humanlike.

Our second hypothesis was that participants would have a harder time distinguishing between human co-players and social AI co-players, and was supported by our findings. Participants' accuracy in detecting human co-players was not significantly above chance, and the level at which the social AI co-player was rated as a human was above the threshold for a typical Turing test. Participants reported no significant differences in how much they perceived human and social AI co-players as a “real person,” and, somewhat surprisingly, the social AI co-player received the highest ratings on this measure. But the social AI's higher rate of perceived humanness was also accompanied by lower ratings of how much participants trusted this co-player compared to human co-players. This relationship may be related to the fact that participants sometimes perceived that the social AI co-player was acting randomly or unpredictably, which is consistent with prior research (Short et al., 2010; Waytz et al., 2010b; Hayes et al., 2014) and this cue was never used to describe any other co-player (see Table 1).

Considering which observed behaviors participants used as cues of humanness in this experiment, the most frequently reported cues were related to whether or not the co-player engaged in interactions with the participant, and the perception that the co-player *did* engage with them was strongly associated with the perception of humanness. This test was solely based on observable non-verbal behavior and not communication, which was disabled. Communication in games through chat is likely the most common type of engagement normally. The fact that many humans did not choose to interact very often did not stop the majority of participants from assuming that a human would try to interact (for instance either expressing that they thought the co-player was not human because they did not interact, or saying they were human because they did interact). This finding is further supported by the fact that, on average, co-players that were perceived as humans engaged participants in social interactions more often than they were engaged by participants. While social AI co-players engaged the participant most often on average, these interactions were sometimes perceived as unpredictable. While the social AI was built upon participants' descriptions of what humanlike motivations and behavior should look like, more training and fine-tuning in how it perceives and responds to social cues would be necessary if such an agent was intended for long-term human-AI social interactions.

It is also somewhat interesting that participants who played with the simple AI co-player had the smallest in-game distance, yet these participants reported that the agent did engage with them less frequently than human co-players and social AI co-players. This may indicate that participants had the opportunity to interact with these agents, yet the overall engagement with the simple AI co-player was low. It is certainly possible that these agents' limited capacity to perceive social actions and social context precluded them from engaging in any meaningful ways with participants, providing more justification to the notion that social AI needs to be equipped with the capacity to sense social cues presented by human interaction partners and respond appropriately. Distance between participants and co-players is not easily interpretable. Close distance provides the opportunity to interact and observe and could have been interpreted as a social signal in and of itself by participants. At the same time, distance may be affected by social interactions, where players who may not trust each other can intentionally choose to distance themselves. Participants who were trying to make a decision on the co-players' identity may also have attempted to stay closer. The only conclusion that might be made is that the navigating behavior of the social AI is more similar to human behavior than that of the simple AI. While movement was not analyzed in this fashion, this is supported by the fact that the social AI was designed to interpret and navigate the game like a human player would, while the simple AI was only designed to perform actions that seemed humanlike without any real motivation to explore. Overall, our results suggest that humans are sensitive to social engagement by co-players within a complex environment, and use this cue to determine whether or not an actor is a human. Our social AI engaged in interact with participants and was perceived as a human more often than simplistic AI, and might be considered to have passed the Turing test, though its perception of the social context and decisions on how to engage may have been perceived as unpredictable and resulted in consequences to how much participants trusted these agents. It is important to note that these results were obtained for one AI system within the context of a videogame, so the generality of such findings for different types of social AI and in different contexts is not known. Videogames provide a great ecologically valid environment to investigate what cues humans make evaluate of AI agents when given flexibility in how to make evaluations. On the other hand, the context can be highly specific, which guides expectations for behavior and makes it necessary to develop AI agents that can meet those specific expectations. However, the game *Don't Starve Together* was selected due to its naturalistic and extremely open environment and participants were allowed to behave and evaluate their co-player however they desired aside from the disabled chat function. The author believes that this lent the experiment ecological validity, which helps to generalize the findings. Furthermore, the social AI was developed to be cognitively plausible, with humanlike motivations that were taken from participants' descriptions of how humans play this type of game, making the social AI generalizable to the context of survival within a naturalistic environment.

Scientists and designers need to improve our understanding of how well people can distinguish between actual humans and current state-of-the-art AI on the basis of behavior within complex environments. As AI begins to incorporate humanlike behavioral traits and motivations, the result may be that the information from these actors is more comprehensible and satisfying (Romero et al., 2017; Ehsan et al., 2018), yet there will be the potential for other humans to use the technology in ways that pose serious threats to our society (Zaleski, 2016; Schwartz, 2018; Huhn, 2019; Wagner and Blewer, 2019).

We believe that these results add to the growing body of literature concerned with the development of artificial social agents, and is aimed at developing a deep understanding of how humans perceive and interact with them. These results imply that an ability to engage socially is perceived as a humanlike quality and suggest that the design of cognitively plausible social AI might help such agents understand how to engage with human interaction partners.

## Data Availability Statement

The datasets generated for this study are available on request to the corresponding author.

## Ethics Statement

The studies involving human participants were reviewed and approved by Institutional Review Board of George Mason University. Written informed consent for participation was not required for this study in accordance with the national legislation and the institutional requirements.

## Author Contributions

ST and EW conceptualized the study and designed the experiment. ST and WK developed the AIs. ST modified the video game and collected the data. ST, WK, and EW analyzed and interpreted the data, theoretically embedded and discussed the results, and wrote the paper. All authors contributed to the article and approved the submitted version.

## Conflict of Interest

The authors declare that the research was conducted in the absence of any commercial or financial relationships that could be construed as a potential conflict of interest.
